# Modeling and Optimization of Pollutants Removal during Simultaneous Adsorption onto Activated Carbon with Advanced Oxidation in Aqueous Environment

**DOI:** 10.3390/ma13194220

**Published:** 2020-09-23

**Authors:** Lidia Dąbek, Anna Picheta-Oleś, Bartosz Szeląg, Joanna Szulżyk-Cieplak, Grzegorz Łagód

**Affiliations:** 1 Faculty of Environmental, Geomatic and Energy Engineering, Kielce University of Technology, Tysiąclecia Państwa Polskiego 7, 25-314 Kielce, Poland; ldabek@tu.kielce.pl; 2 Department of Environment and Waste Management, Marshal’s Office of the Świętokrzyskie Voivodeship, IX Wieków Kielc 3, 25-516 Kielce, Poland; anna.picheta-oles@sejmik.kielce.pl; 3 Faculty of Fundamentals of Technology, Lublin University of Technology, Nadbystrzycka 38, 20-618 Lublin, Poland; j.szulzyk-cieplak@pollub.pl; 4 Environmental Engineering Faculty, Lublin University of Technology, Nadbystrzycka 40B, 20-618 Lublin, Poland

**Keywords:** modelling and optimization, wastewater purification, activated carbon, sorption, advanced oxidation

## Abstract

The paper presents the results of studies on the modeling and optimization of organic pollutant removal from an aqueous solution in the course of simultaneous adsorption onto activated carbons with varied physical characteristics and oxidation using H_2_O_2_. The methodology for determining the models used for predicting the sorption and catalytic parameters in the process was presented. The analysis of the influence of the sorption and catalytic parameters of activated carbons as well as the oxidizer dose on the removal dynamics of organic dyes-phenol red and crystal violet-was carried out based on the designated empirical models. The obtained results confirm the influence of specific surface area (S) of the activated carbon and oxidizer dose on the values of the reaction rate constants related to the removal of pollutants from the solution in a simultaneous process. It was observed that the lower the specific surface area of carbon (S), the greater the influence of the oxidizer on the removal of pollutants from the solution. The proposed model, used for optimization of parameters in a simultaneous process, enables to analyze the effect of selected sorbents as well as the type and dose of the applied oxidizer on the pollutant removal efficiency. The practical application of models will enable to optimize the selection of a sorbent and oxidizer used simultaneously for a given group of pollutants and thus reduce the process costs.

## 1. Introduction

The increasing population, environmental pollution and climate change effects, such as severe droughts, place a heavy burden on water resources. Industry is the sector which exerts the strongest environmental pressure on water quality. Along with its intensive development, increasing amounts of new and complex chemical compounds occur, which together with wastewater, as well as municipal and industrial wastes, infiltrate into the natural environment, usually in the first stage during migration to surface waters [1,2]. Since industrial wastewater is characterized by heterogeneous composition and the content of numerous pollutants with a broad spectrum of physicochemical properties (toxic compounds, dyes, pigments, heavy metals), their efficient treatment requires the application and combination of numerous methods [3]. In this aspect, the results of the latest studies indicate the great potential of technologies based on the process synergy [4,5,6,7,8], among which much attention is devoted to the application of sorption with activated carbon as sorbent and the advanced oxidation methods [9,10,11,12,13,14,15]. Both adsorption [16,17,18] and advanced oxidation processes (AOPs) [19,20,21,22] are efficient methods for removing hardly-biodegradable compounds from wastewater, however, these technologies have certain limitations. The popularization of the adsorption processes is hindered by the high costs of the applied adsorbents as well as the issues with the regeneration or disposal of the adsorbents spent in the treatment process. In turn, the advanced oxidation methods often require the use of large doses of oxidizers and specialized, expensive devices, including ozone and ultrasound generators, or ultraviolet (UV) lamps. The integration of both processes may significantly mitigate the aforementioned drawbacks. In this case, the removal of pollutants is carried out either as a single or a two-step process. In the former, the removal of pollutants involves sorption and then oxidation resulting in a simultaneous regeneration of the activated carbon. In the latter, simultaneous sorption and oxidation of organic compounds occurs. Such a solution is justified, and the research results indicate that the integration of adsorption with advanced oxidation processes may significantly improve the efficiency and shorten the treatment process, as well as reduce the costs of the conducted technological process [23,24,25]. Additionally, the advanced oxidation processes can be employed for the degradation of the adsorbed organic pollutants, which simultaneously leads to the adsorbent regeneration [26,27].

The literature data [28,29,30,31] indicate that activated carbons catalyze the decomposition of oxidizers, such as hydrogen peroxide or ozone, generating highly reactive hydroxyl radical in the process. This reaction may be successfully used for the oxidation of organic pollutants in aqueous solutions. The system comprising activated carbon-oxidizer-organic pollution, probably involves the sorption and catalytic oxidation processes.

Despite numerous studies, the course of this process is not explained in a clear way. According to Toledo [30], the organic compounds are first adsorbed onto the activated carbon and then oxidized, whereas Vidic [32] believes that the catalytic role of the activated carbon in the creation of hydroxyl radicals in the reaction environment is the most important. Sanchez-Polo et al. [33,34] and Alvarez et al. [35] reported that activated carbon acts as a promoter for ozone oxidation, rather than a true catalyst, because it is modified during the ozonation process. The results obtained by Dąbek et al. [36] confirm that the decomposition of hydrogen peroxide and creation of hydroxyl radicals responsible for the oxidation of dyes occur in the presence of the activated carbon. The concentration of the oxidizer in the solution has to be matched to the amount of the oxidized substances in order to avoid the suppression of radicals. Santos et al. [37] indicated that the chemism of the activated carbon surface affects the course of this process. Similar conclusions were drawn by Malaika et al. [38] and Pereira et al. [39], who indicated that both the sorption and catalytic capacities of carbon xerogel (CX) depend significantly on the chemical structure of its surface. In turn, Faria et al. [26] noted that the activated carbon is characterized not only by high adsorption capacity but also catalytic properties due to high specific surface. Huang et al. [31] compared several activated carbon samples before and after processing with the oxidizing agents, which increased the content of acidic surface functional group on the activated carbon. The authors observed that the decomposition activity of the modified activated carbon sample was lower in relation to hydrogen peroxide, whereas the catalytic activity in relation to the decomposition of 4-chlorophenol was slightly higher than when fresh activated carbon was used.

The results of previous research indicate that the removal of organic compounds from aqueous solution using activated carbons and oxidizing agents belonging to AOPs is a more efficient process than in the case where the oxidation and sorption processes are realized separately. However, there is no universal treatment technology using simultaneous processes. In each case, the technology is adjusted to the specific properties of wastewater to be treated. The opinions on the mechanics of this process are mixed. There is no information on the properties that should characterize the activated carbons used in these reactions and what is the influence of the oxidizing agent on the physicochemical properties of activated carbons. As indicated in previous studies [14], the efficiency of pollution removal in simultaneous adsorption and advanced oxidation processes is governed by a broad range of parameters, the most relevant of which include the sorption and catalytic properties of the adsorbent. These parameters largely determine the process efficiency as well as the possibility of applying a sorbent under specific conditions.

The application of simultaneous methods for their optimization enables to analyze the influence of the properties of selected sorbents as well as the type and dose of the applied oxidizer on the efficiency of pollutant removal.

The application of mathematical models for predicting the course of the adsorption process with set operational sorption parameters was investigated in numerous papers [40,41,42,43]. The literature sources present numerous methods and mathematical models based on the kinetics and adsorption isotherms enabling to predict the process. The most commonly used kinetic expressions to explain the solid/liquid adsorption processes are the pseudo-first-order kinetics and pseudo-second-order kinetic models [44,45,46,47]. This approach enables modeling the process dynamics in time. Due to the high costs of conducting experiments, the methods which allow for decreasing the number of measurements, i.e., design of experiments (DoE) are highly useful. Response surface method (RSM) can be mentioned as an example [48]. The DoE methods reduce the number of necessary measurements, which shortens the required time, decreases the material consumption and the costs of experiments [49,50,51]. Due to the limited application of the models designated by means of RSM, the models devised with the data mining methods, for instance artificial neural networks (ANN), are highly useful [52,53,54].

However, in a majority of cases, the models presented describe the sorption of single pollutants on selected carbon adsorbents. Moreover, greater attention was drawn to the sorption process conditions, rather than to the physicochemical properties of the employed activated carbon. As a result, the practical application of such models is limited, because they cannot be used for predicting sorption of another group of adsorbents. No information on the modeling of sorption in simultaneous processes in the presence of oxidizers was found in the literature. Therefore, devising the models for predicting sorption on activated carbons, which account for the diversity of the physicochemical parameters of a sorbent, different adsorbates and the presence of an oxidizing medium, seems to be reasonable.

The paper presented the methodology of creating the mathematical models for optimization of a simultaneous sorption process of activated carbon adsorption with chemical oxidation of pollutants from aqueous solutions via hydrogen peroxide, on the example of crystal violet and phenol red. The article showed the methodology of conducting statistical analyses aimed at determining the influence of selected carbon characteristics (dechlorination half-length (D_ehl_), methylene number (MBN), iodine number (IN), S, soluble substance content (Cont.s.sol)) and oxidizer dose (H_2_O_2_) on the dynamics of the sorption process (Freundlich and Langmuir isotherms) and removal of pollutants from aqueous solutions (reaction rate constants in pseudo-first- and pseudo-second-order equations). The obtained results were used to designate mathematical models (by means of the multiple regression method) for calculating the parameters describing the dynamics of sorption and pollutant removal based on the activated carbon characteristics and oxidizer dose. The calculations were performed using the results of laboratory studies as well as identification of sorption and pollutant removal dynamics in aqueous solutions [36]. The obtained dependencies enable modeling the above-mentioned processes with diversified characteristics and equal oxidizer doses. The obtained models enable optimizing the selected parameters of the simultaneous process of pollutant removal (crystal violet and phenol red) from aqueous solutions by adjusting the oxidizer dose that enables to achieve shortest pollutant reduction time, depending on the activated carbon used.

The practical application of the devised models will enable to optimize the selection of the sorbent and the oxidizer used simultaneously for a given group of pollutants and thus will reduce the process costs of their removal from aqueous environment.

## 2. Materials and Methods

### 2.1. Materials

The object of research involved the two dyes: crystal violet (C_25_H_30_CIN_3_) and phenol red (C_19_H_14_O_5_S), commonly used for dyeing fabrics, paper products and printing ink, thus found in the textile and dye industry wastewater. They are also used as analytic reagents. These substances are characterized by low efficiency of removal from aqueous solutions and wastewater using physical and biological methods.

In the research, H_2_O_2_, which is found in the wastewater from the textile, dye, and printing industries, was employed as the oxidizing agent [55].

In order to determine the correlations between the activated carbon properties and the removal efficiency of the analyzed organic substances, the following organic carbons were selected (see Table S1 Supplementary Materials):

- WDex and WG-12–fresh commercial granulated activated carbons produced in Poland by Gryfskand (Gryfino), used in the treatment of water and wastewater, removal of organic compounds, including pesticides, as well as dechlorination of water and improvement of its taste;
- F-200S–spent F-300 carbon following several years of use in a water treatment station in Goczałkowice, to be disposed of;
- F-200R–F-200S activated carbon following several years of use in a water treatment station in Goczałowice, subsequently regenerated with the Fenton’s agent.

### 2.2. Methods

**Figure 1:**
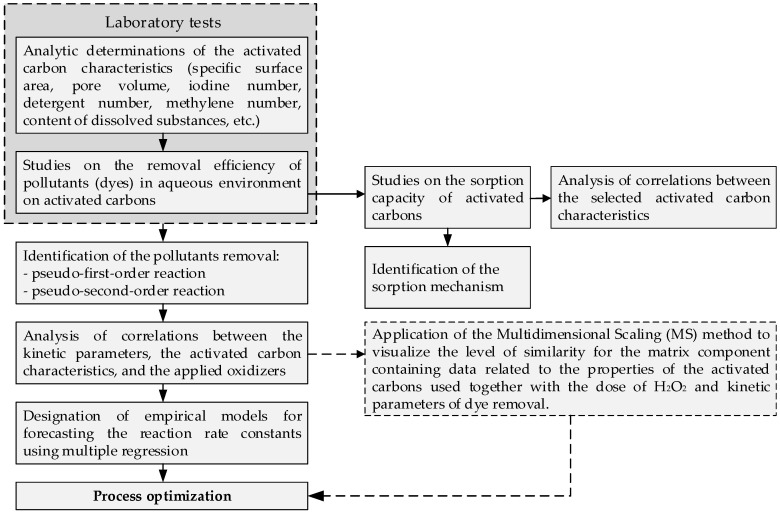
Algorithm for the creation of a model predicting the kinetics of sorption and pollutant removal.

The methodology for the designation of models predicting the kinetic parameters in the models describing the sorption isotherms and removal of pollutants in aqueous solutions using activated carbons, was proposed (Figure 1).

The analyses in the assumed methodology are based on the results of laboratory tests related to sorption and pollutant removal performed on the activated carbons with diversified physical characteristics (specific surface area-S, pore volume-V_p_, dechlorination half-length-De_hl_, iodine number-IN, detergent number-DetN., methylene number-MBN, ash content-Cont.ash, soluble substance content-Cont.s.sol., pH of the aqueous extract-pH) with hydrogen perodixe (H_2_O_2_) as the oxidizer.

#### 2.2.1. Methodology of Laboratory Tests

**Figure 2:**
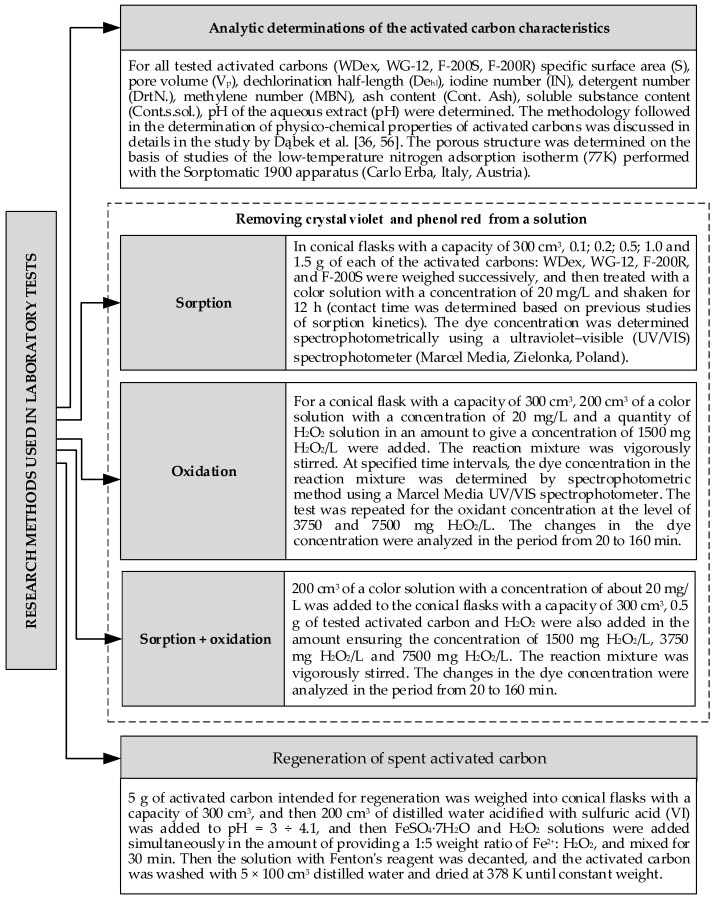
Methodology of laboratory studies [36,56].

The measurement experiments can be divided into several stages which involve analytic determination of the activated carbon characteristics, removing crystal violet and phenol from a solution through: sorption, oxidation via H_2_O_2_ and a simultaneous process (sorption + oxidation with H_2_O_2_) as well as regeneration of spent activated carbon. The subsequent measurement steps and their methodology are discussed in detail in Figure 2.

#### 2.2.2. Identification of the Pollutant Removal

On the basis of the conducted experiments (sorption of pollutants onto activated carbon, removal of pollutants from the solution), the empirical models were fitted to the theoretical data. For the sorption models, the process kinetics parameters were determined based on the Langmuir and Freundlich isotherms. While removing pollutants from the solution (activated carbon as the oxidizer), the process kinetics was identified based on the pseudo-first- and second-order equations. Thus, the values of reaction rate constants were determined.

The influence of carbon characteristics and the oxidizer dose on their dynamics was analyzed on the basis of the process kinetics calculations (sorption, removal of pollutants from the solution). For this purpose, the correlation matrices were calculated. This enabled to determine which parameters of activated carbons and oxidizers affect the sorption and pollutant removal processes. Moreover, the correlation between the analyzed characteristics of activated carbons, having a significant influence on the selection of optimal independent variables for the kinetic models, was determined. This served as the basis for designating the models for predicting the values of process kinetics parameters. The characteristics of activated carbons were optimized in order to achieve high pollutant removal efficiency in shortest time possible. The proposed solution, in contrast to those presented in other papers [57,58,59] is universal, because it enables to analyze the influence of the analyzed sorbent (here: activated carbon) and employed oxidizer as well as their properties. The previous models usually did not account for these aspects and considered only a single activated carbon, which led to their numerous limitations.

The analysis of sorption onto activated carbons was performed assuming the following equations:

- Freundlich:

(1) $$q_{e}=K_{F}\cdot c_{e}{}^{\frac{1}{n}}$$

- Langmuir:

(2) $$q_{e}=\frac{a_{m}\cdot K_{L}\cdot c_{e}}{1+K_{L}\cdot c_{e}}$$

where q_e_—amount of adsorbate adsorbed per unit mass of activated carbon (mg/g), c_e_—equilibrium concentration of the adsorbate (mg/L), K_F_—coefficient in the Freundlich isotherm model (mg/g), n—coefficient in the Freundlich isotherm model, K_L_—coefficient in the Langmuir isotherm model (L/mg), a_m_—sorption capacity value in the Langmuir isotherm model (mg/g).

On the basis of the studies on the changes in the concentration of crystal violet and phenol red in the solution in the presence of activated carbon and oxidizer [33] the following kinetic equations were assumed:

- Pseudo-first-order:

(3) $$\log\left(c_{0}-c_{t}\right)={\mathrm{logc}}_{0}-\frac{k_{1}\cdot t}{2.303}$$

- Pseudo-second-order:

(4) $$\frac{1}{c_{t}}=\frac{1}{k_{2}\cdot c_{0}^{2}}+\frac{1}{c_{0}}\cdot t$$

where c_0_—initial substrate concentration (mg/L), c_t_—substrate concentrations after a given time (t) (mg/L), t—reaction time (s), k_1_—first order reaction rate constant (s^−1^), k_2_—second order reaction rate constant (M^−1^·s^−1^)

In the case of sorption and pollutant removal from the solution, the correlation between the results and calculations was measured using the coefficient of determination (R^2^). On the basis of the obtained value of this parameters, the identified sorption mechanisms (process physics) were discussed for the analyzed variants of the experiments (activated carbon parameters, oxidized dose) in terms of the designated R^2^ values and differences between them. For instance, when the calculated R^2^ value for the data interpolated using a pseudo-second order equation was greater than the data modeled with a pseudo-first ordered equation, it was assumed that the kinetics of pollutant removal was in line with Equation (4). In turn, when the R^2^ value obtained for the data interpolated with the Freundlich isotherm equation was greater than the results of calculations using the Langmuir isotherm, it was assumed that the sorption mechanism was in accordance with relation Equation (1).

#### 2.2.3. Multiple Linear Regression

Multiple linear regression (MLR) constitutes one of the simplest and most commonly employed methods for creating linear models. In this method, the forecast relationship has the following form [60]:

(5) $$y=\overset{M}{\underset{i=1}{\sum }}a_{i}\cdot x_{i}$$

where a—model parameters determined with the least squares method, x—input variables (independent), y—model output, i—number of independent variables (i = 1, 2, 3, …, M).

In order to optimally assign the independent variables to the empirical models, the correlation coefficients were calculated between the characteristics of carbons as well as the kinetics parameters in the sorption models (Freundlich and Langmuir isotherms) and pollutant removal (dyes-crystal violet, phenol red). These calculations enabled to eliminate the dependent variables from the models. By applying the multiple regression method, the empirical models for calculating the coefficients pertaining to sorption (K_L_, a_m_, K_F_, and n) as well as removal of pollutants from the solution (k) were determined.

#### 2.2.4. Optimization of Pollutant Removal from the Solution

During the process design, the costs of reagents, its dynamics and degree of pollutant reduction in wastewater are of great importance. In order to design a process optimally, the costs of reagent consumption have to be decreased while simultaneously achieving the highest reduction of pollutants in shortest time possible. On the basis of Equations (3) and (4), the subsequent conditions for process optimization were described using the following relationships:

- Pseudo-first-order:

(6) $$\{\begin{array}{c} t=\frac{2.303\cdot \log\left(\frac{1}{1-\beta }\right)}{k_{1}}\\ k=f\left(S,\;H_{2}O_{2},\;\ldots .,x_{i}\right)\\ t\to \min \end{array}$$

- Pseudo-second-order:

(7) $$\{\begin{array}{c} t=\frac{1}{k_{2}}\cdot \left(\frac{k_{2}\cdot c_{0}}{\beta }-1\right)\\ k=f\left(S,\;H_{2}O_{2},\;\ldots .,x_{i}\right)\\ t\to \min \end{array}$$

where β—degree of pollutant removal expressed as c_t_ c_0_^−1^; c_0_—in line with the research methodology (mg/L), the value c_0_ = 20 mg/L was assumed in the calculations for phenol red and crystal violet; c_t_—substrate concentrations after a given time (t) (mg/L).

In the presented analyses, the main criterion for the optimization of pollutant removal from wastewater was time t, which enabled to achieve high reduction of dye concentration in the aqueous solution. Therefore, the combination of carbon characteristic and oxidizer dose, for which time t enabling high reduction of pollutants (η = 90%) in the solution was the shortest, was sought.

On the basis of Equations (6) and (7), the influence of activated carbons characteristics and oxidizer doses on shortening the time of pollutant removal from the solution (crystal violet and phenol red) was analyzed assuming the required reduction.

#### 2.2.5. Visualization of Data with Multidimensional Scaling Method

The multidimensional scaling (MS) method was used in order to visualize the similarity level for the component of matrix containing the data connected with the properties of employed activated carbons along with the H_2_O_2_ dose and the kinetic parameters of dye removal. The MS method [61,62] made it possible to obtain sets of points distributed in a 2-dimensional space in such a way that in the analyzed cases (tested activated carbons in different versions of the experiment), the similar points were closest to each other, while different ones were spread far apart [63]. Its aim was to show in one figure the general similarity between the processes carried out with the use of four activated carbons (WDex, WG-12, F-200R, F-200S) characterized by different properties (S, IN, MBN, De_hl_), using different doses of the oxidizer (0, 1500, 3750, 7500 mg H_2_O_2_/L), which were associated with obtaining different kinetic constants k_1_ and k_2_ with respect to the removal of both analyzed dyes from the water solution (crystal violet, phenol red).

## 3. Results and Discussion

### 3.1. Identification of the Sorption Process

On the basis of the conducted research (Figure 1), the theoretical curves were matched to the empirical data. The calculation results for sorption were presented in Table 1, whereas for the removal of pollutants in the presence of crystal violet and phenol red-in Table 2. Taking into account the data presented in Table 1, the sorption mechanism of the analyzed dyes cannot be unequivocally determined for all activated carbons. In the case of fresh carbons (WDex, WG-12), the sorption process dynamics of the crystal violet solution is in line with the Langmuir isotherm. However, for phenol red, the situation is not as clear-in the case of WDex, the coefficient of determination R^2^, similarly as for crystal violet, is higher for the Langmuir model, whereas for WG-12, the R^2^ coefficients are at a similar level. In turn, for the spent activated carbons (F-200R and F-200S) it can be stated that in the case of the regenerated spent carbon F-200S, the sorption dynamics is clearly in accordance with the Freundlich isotherm for both considered dyes. However, the match is not as obvious for spent carbon F-200R, the coefficients of determination R^2^ are at a similar level in relation to both models; however, for phenol red, R^2^ is greater for the Langmuir model. Due to slight differences between the coefficients of determination R^2^, in majority of cases it was difficult to unequivocally determine the sorption mechanism, which was discussed in detail in the paper by Dąbek et al. [36]. While analyzing the literature data, it was stated that most authors, in relation to the sorption of dyes onto activated carbon, pointed at the monolayer sorption mechanism [64,65,66,67].

The obtained sorption results indicate that in the case of the fresh activated carbons, this process can be employed as a method for the removal of the analyzed dyes; however, this process requires longer time and is difficult to implement in an industrial installation, due to the presence of other adsorbing substances and suspensions.

### 3.2. Removal of Pollutants from the Solution (Cystal Violet and Phenol Red)

As indicated by the abovementioned results, the sorption process efficiency can be improved by introducing an oxidizing agent into the system. The literature review showed that although the authors unequivocally indicate the purposefulness of integrating sorption and AOPs in order to improve to efficiency of pollutant removal, the mechanism of the simultaneous process is not fully explained; therefore, in order to ensure the optimal conditions, it is necessary to correlate the parameters related to the sorbent parameters as well as the type and dose of the oxidizer. On the basis of Equations (3) and (4), the reaction rate constants in the dye decomposition processes (crystal violet and phenol red) in the presence of an oxidizer (H_2_O_2_), were determined. The calculation results were presented in Table 2.

### 3.3. Analysis of Correlation between the Activated Carbon Characteristics

In order to comprehensively evaluate the relationships between the particular activated carbon parameters, a Spearman’s rank correlation coefficient was prepared (Table 3), which indicates that between the dechlorination half-length iodine number, ash content and pH of the aqueous extract occurs at the confidence interval of p = 0.05. In Table S2 in Supplementary Materials determined values of the test probability are given for the correlation between the characteristics of the activated carbons.

It should be noted that an increase in the pH of the aqueous extract and the iodine number decreases the dechlorination half-length, i.e., the dechloration ability increases, which indirectly indicates enhanced catalytic properties of carbon in the reaction involving decomposition of hydrogen peroxide with the formation of hydroxyl radicals [14]. A strong correlation between the iodine number as well as the methylene number and specific surface area is observed as well. A similar correlation was indicated by Nunes et al. [68], who stated that the iodine number and methylene blue number are important for the surface area estimation of the activated carbons, since the materials possess different pore sizes which can be accessed by the different molecules according to the pore geometry. Moreover, while analyzing the obtained correlation matrix (Table 3), correlations between specific surface, pore volume, pH of the aqueous extract and ash content, were noted.

### 3.4. Influence of Carbon Characterisitcs on the Parameters in the Models for Sorption and Pollutant Removal

#### 3.4.1. Adsorption onto Activated Carbons

In order to complete the analyses, the possibility of determining the values of the model parameters (K_F_ and n in the Freundlich isotherm model, a_m_ and K_L_ in the Langmuir isotherm model) using empirical models was considered. This aspect is very important from the point of generalizing the calculation results and creating universal models. Taking into account the correlation of selected characteristics of activated carbons (Table 3) and the different sorption process course (crystal violet, phenol red), Table 4 presents examples of relationships K_F_ = f(x_i_) and n = f(x_i_) as well as a_m_ = f(x_i_) and K_L_ = f(x_i_).

Taking into account the results of calculations (Table 3), the investigations were narrowed down to the analysis of independent variables. Table 4 presents the dependencies for which the coefficients of correlation and determination are high.

The results presented in Table 4 indicate a strong correlation between the specific surface area (S), and K_F_ and n, as well as between the content of dissolved substance (Cont.s.spl.) and n. The obtained relations between the parameters in the Freudlich and Langmuir models confirm in most cases that it is possible to develop universal models in which the kinetics of the sorption process is estimated based on the characteristics of the carbons.

#### 3.4.2. Removal of Pollutants from Aqueous Solutions

##### Designation of Empirical Models for Determining the Reaction Rate Constant k

Correlation matrices were created first as part of the conducted analyses, in order to evaluate the influence of selected activated carbon characteristics (WDex, WG12, F200R, F200S) on the concentration of selected pollutants. The results of analyses were presented in Table 5 and Table 6. The conducted analyses (Table 5) in the confidence interval of *p* = 0.05 indicated a strong correlation between the mean dye concentration and the dechloration number, iodine number, as well as specific surface area. In Table S3 in Supplementary Materials determined values of the test probability are given for the correlation between the selected characteristics of the activated carbons and average dye concentration (c_av_).

The correlation matrix also indicates that the greater the dechloration number (lower catalytic capacity of the activated carbon), the greater the mean dye concentration in the solution following the process.

Due to the high costs of the studies and the time of conducting the supplementary determinations, an attempt was made to designate the empirical models for predicting the reaction rate constants (k) in the pseudo-first (or second) order equations. For this purpose, the correlation matrices reflecting the relations between the determined kinetic parameters and the characteristics of carbons and the oxidizer dose, were designated. Then, depending on the obtained values of correlation coefficients, the empirical models were determined. The multiple regression method was employed for this purpose.

The identification of independent variables for the model was performed using the Fischer-Snedecor distribution model. Statistically significant variables, in the confidence interval of 0.05 were assumed for analyses. Due to the limited amount of measurement data, the multiple cross validation method was used for the numerical experiment. The values of correlation coefficients for crystal violet and phenol red were given in Table 7 and Table 8, respectively. In Table S4 in Supplementary Materials determined values of the test probability are given for the correlation between the selected characteristics of the activated carbons and the reaction rate constants in the pseudo-first and pseudo-second order equations.

The conducted analyses (Table 7 and Table 8) indicated that:

- There is a correlation (r > 0.5) between the pseudo-first (second) order kinetic parameters and specific surface area (S), methylene number (MBN), dechlorination half-length (De_hl_) and oxidizer dose (H_2_O_2_).
- There is almost complete correlation (r > 0.9) between the pseudo-first (second) order kinetic parameters and specific surface area (S), methylene number (MBN) and dechlorination half-length (De_hl_).
- An increase in S, De_hl_, and H_2_O_2_ dose leads to an increase in the values of kinetic parameters (k_1_, k_2_) of pseudo-first(second) order.

On the basis of the above-mentioned analyses, independent variables for the models used to calculate the k_1_ and k_2_ values were determined with the Fischer-Snedecor distribution method. This served as a basis for creating the empirical models using the MLR method, having the following form:

- crystal violet (pseudo-first order):

(8) $$k_{1}=0.000023\cdot S+0.000002\cdot H_{2}O_{2}-0.0026\cdot {\mathrm{De}}_{\mathrm{hl}}\;\;\;R^{2}=0.79$$

- crystal violet (pseudo-second order):

(9) $$k_{2}=0.000057\cdot S+0.000007\cdot H_{2}O_{2}-0.0083\cdot {\mathrm{De}}_{\mathrm{hl}}\;\;\;R^{2}=0.69$$

- phenol red (pseudo-first order):

(10) $$k_{1}=0.000016\cdot S-0.00169\cdot {\mathrm{De}}_{\mathrm{hl}}.+0.000001\cdot H_{2}O_{2}\;\;\;R^{2}=0.92$$

- phenol red (pseudo-second order):

(11) $$k_{2}=0.000033\cdot S-0.00389\cdot {\mathrm{De}}_{\mathrm{hl}}+0.000001\cdot H_{2}O_{2}\;\;\;R^{2}=0.89$$

##### Optimization of Pollutant Removal from the Solution

The influence of the activated carbon characteristics as well as the activated carbon characteristics and the oxidizer dose on the dynamics of pollutant removal from the aqueous solution was investigated. In the case of the analyzed dyes, higher coefficients of correlation were obtained using the pseudo-second order reactions (see Table 2). Therefore, it was assumed that the removal of phenol red and crystal violet from aqueous solutions are described by the pseudo-second order reaction. Therefore, while determining the pollutant removal time, only Equation (7) were used, whereas the empirical models for indicating the reaction rate constants were based on Equations (9)-crystal violet and (11)-phenol red. The influence of carbon characteristics and carbon dose on the pollutant removal dynamics was investigated using the following relationships:

(12) $$\eta_{t\left(1\right)}=\frac{t\left(S=S_{0}+\Delta S,{\;H}_{2}O_{2,\;}\;\beta \right)}{t\left(S=S_{0},H_{2}O_{2},\;\beta \right)}$$

(13) $$\eta_{t\left(2\right)}=\frac{t\left(S=S_{0}+\Delta S,\;H_{2}O_{2,\;}\;\beta \right)}{t\left(S=S_{0}+\Delta S,\;\beta \right)}$$

(14) $$\eta_{t\left(3\right)}=\frac{t\left(S=S_{0}+\Delta S,{\;H}_{2}O_{2,\;}\;Z.\mathrm{De}.={\mathrm{De}}_{\mathrm{hl}}._{0}+Z.\mathrm{De}.,\Delta \beta \right)}{t\left(S=S_{0}+\Delta S,H_{2}O_{2,\;}{\mathrm{De}}_{\mathrm{hl}}._{0},\;\beta \right)}$$

where S_0_—minimal surface of activated carbon assumed for calculations; here, it amounts to S_0_ = 750 m^2^, ∆S—calculation step assumed in the analysis pertaining to the impact of changes in the specific surface of activated carbon on the reduction of pollutants; in this work, it was assumed as ∆S = 25 m^2^, H_2_O_2_-oxidizer dose, assumed as 0–3500 mg; β—degree of pollutant reduction in the solution, assumed as 90%.

Equation (12) enables one to analyze the influence of increasing specific surface of activated carbon for different assumed oxidizer doses on the changes in the pollutant removal time for the required reduction degree. In turn, Equation (13) allows analyzing the influence of increasing the oxidizer dose for the assumed surfaces of activated carbons on shortening the removal time of pollutants from the aqueous solution until the assumed reduction degree—β = c_t_·c_0_^−1^—is achieved. On the basis of Equation (14) it is possible to determine the effect of changes in the De_hl_ value on the time of pollutant removal from the solution at the assumed S values and H_2_O_2_ dose.

**Figure 3:**
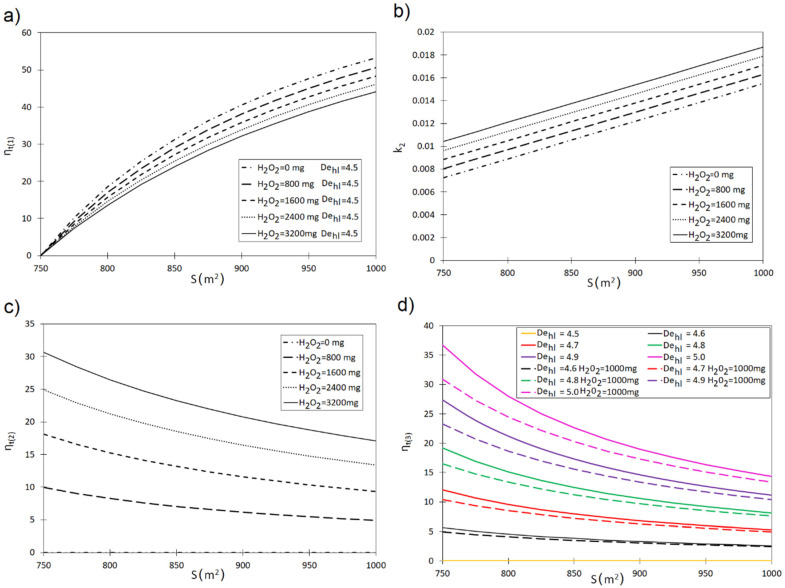
(**a**) Influence of specific surface of activated carbon (S) and oxidizer dose (H_2_O_2_), for De_hl_ = 4.5 on η_(1)_. (**b**) Influence of specific surface of activated carbon (S) and oxidizer dose (H_2_O_2_) for De_hl_ = 4.5 with reaction rate constant k_2_ in pseudo-second order equation. (**c**) Influence of specific surface of activated carbon (S) and oxidizer dose (H_2_O_2_) for De_hl_ = 4.5 on η_(2)_. (**d**) Influence of specific surface of activated carbon (S), oxidizer dose (H_2_O_2_), and dechlorination half-length (De_hl_)on η_(3)_.

**Figure 4:**
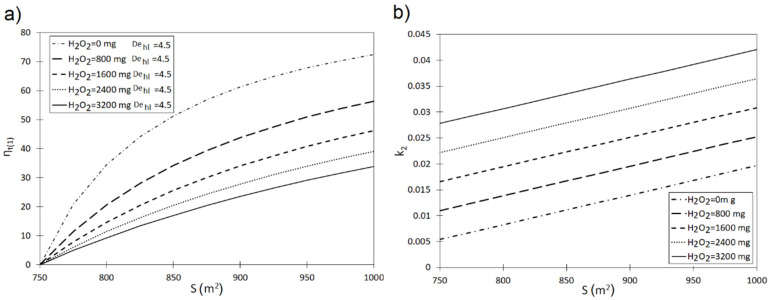

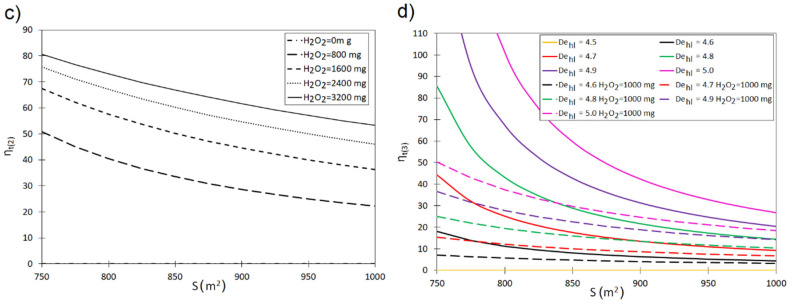
(**a**) Influence of specific surface of activated carbon (S) and oxidizer dose (H_2_O_2_), for De_hl_ = 4.5 on η_(1)_. (**b**) Influence of specific surface of activated carbon (S) and oxidizer dose (H_2_O_2_) for De_hl_ = 4.5 with reaction rate constant k_2_ in a pseudo-second order equation. (**c**) Influence of specific surface of activated carbon (S) and oxidizer dose (H_2_O_2_) for De_hl_ = 4.5 on η_(2)_. (**d**) Influence of specific surface of activated carbon (S), oxidizer dose (H_2_O_2_), and dechlorination half-length (De_hl_) on η_(3)_.

Equations (11)–(14) were used to designate the η_t(1)_, η_t(2)_ and η_t(3)_ curves describing the influence of activated carbon characteristics and oxidizer dose on the removal of the investigated pollutants from the solution (Figure 3 and Figure 4). The obtained results indicate a significant influence of specific surface of carbon on the efficiency of dye removal. For instance, increasing the S value from 750 m^2^ to 850 m^2^ assuming the H_2_O_2_ dose of 800 mg, shortens the pollutant removal time by 29.10% in the case of phenol red (90% reduction) (Figure 3a). Increasing the oxidizer dose to H_2_O_2_ = 3200 mg indicates that the increase in S within the range of 750–800 m^2^ shortens the minimum time required to achieve the assumed dye reduction level by 24.01%. Identical relations were between the analyzed variables η_t(1)_ = f(S, H_2_O_2_) were determined for crystal violet (Figure 4a). For example, an increase in the carbon surface from S = 750 m^2^ to S = 850 m^2^ at the oxidizer dose of H_2_O_2_ = 800 mg shortens the time required to achieve 90% pollutant reduction by 34.13%. An identical change of S for the H_2_O_2_ dose of 3200 mg shortens that time by 17.01%.

On the basis of the obtained η_t(2)_ curves, it was observed that introducing an oxidizer into the system leads to an increase in the reaction rate constants. For De_hl_ = 4.5 and S = 850 m^2^ the reaction rate constant amounts to k_2_ = 0.0105 (phenol red)-Figure 3b and k_2_ = 0.011 (crystal violet)—Figure 4b. An oxidizer addition in the amount of H_2_O_2_ = 1600 mg increases their values to k_2_ = 0.012 and k_2_ = 0.023, respectively.

This translates into a shortening of the pollutant removal time required to achieve the assumed reduction degree. For instance, inclusion of 800 mg H_2_O_2_ in the reaction system (S = 850 m^2^ and De_hl_ = 4.5) shortens the pollutant removal time (phenol red) by 7% in comparison to the system without an oxidizer (Figure 3c). Assuming S = 850 m^2^, introduction of H_2_O_2_ = 1600 mg into the system shortens the dye removal time (90% elimination) by 33.5% compared to the non-modified process. This is in line with the results obtained by Santos et al. [37], who indicated that a synergistic effect occurs in the case of simultaneous sorption onto activated carbon and oxidation via H_2_O_2_, which enhances the solution decolorization.

While analyzing the obtained η_t(2)_ curves, it was observed that the lower the specific surface of activated carbon (S), the greater the influence of oxidizer on the pollutant removal process. For example, in the case of S = 800 m^2^ and H_2_O_2_ = 1600 mg, 90% crystal violet removal can be achieved 57.0% faster than in the system without this compound. In turn, for S = 900 m^2^, the same pollutant removal effect can be achieved 44.53% quicker. While analyzing the removal of phenol red from the solution, it was observed that for S = 800 m^2^ and H_2_O_2_ = 1600 mg, the 90% removal can be obtained 15.24% faster than in the system without an oxidizer. In turn, for S = 900 m^2^ and H_2_O_2_ = 1600 mg, 90% phenol red removal is 11.60% quicker than without the oxidizing compound.

On the basis of the performed analyses, the η_t(3)_ = f(S, De_hl_, H_2_O_2_) curve was prepared for crystal violet (Figure 4d) and phenol red (Figure 3d). The curve obtained in this way reflects the relative extension of the pollutant removal time enabling to achieve the assumed removal degree. Thus, it can be stated that an increase in De_hl_ decreases the dynamics of pollutant removal. For example, for S = 850 m^2^ the increase in De_hl_ by 4.5–4.7 extends the time required for 90% crystal violet removal by 17.58%. In turn, for S = 850 m^2^ the increase in De_hl_ by 4.5–4.7 extends the 90% phenol red removal time by 7.96%. The obtained curves (Figure 3d and Figure 4d) also confirm that specific surface of activated carbon and oxidizer dose constitute key factors affecting the removal of pollutants from a solution in a simultaneous process. This is in line with the findings of Faria et al. [26], who indicated that activated carbon is characterized not only by high adsorption capacity, but also the catalytic properties, owing to its high specific surface area.

#### 3.4.3. Multidimensional Scaling (MDS)

**Figure 5:**
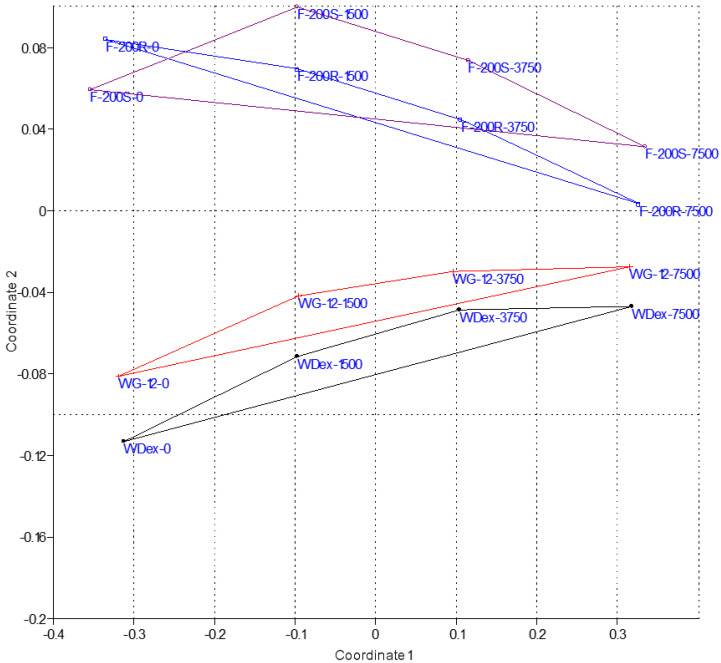
Multidimensional scaling results of activated carbon parameters, together with influence of added H_2_O_ll_ on kinetic parameters of dyers removal from water solution.

The visualization of similarity levels for the matrix containing the data connected with the properties of the applied activated carbons with H_2_O_2_ and the kinetic parameters for the removal of dyes based on the multidimensional scaling (MDS) method was presented in Figure 5.

There is a visible similarity between the fresh activated carbons WG-12 and WDex and between the spent carbons F-200S and F-200R. Taking into account the amount of added H_2_O_2_ oxidizer, it can be seen that the points in the graph for particular carbons move closer to each other as the dose increases, and the increasing similarity of the spent carbon to the general characteristics describing the fresh carbon is much more evident in the case of F-200R. Another similarity presented graphically using Multidimensional Scaling, is that the highest applied oxidizer dose (7500 mg H_2_O_2_/L) causes that the similarities between the analyzed results of dye removal by all employed carbons (WDex, WG12, F-200S, F-200R) are much greater (the points are spread apart) than in the case of the similarity between the employed carbons with a high oxidizer dose and without oxidizer. This is most evident on the example of relatively close proximity of the points corresponding to WDex 7500 and F 200S 7500 compared to the long distance between WDex 7500 and WDex 0. The distance between F-200R 7500 and F-200R 0 is even greater, whereas the point marked as F-200R 7500 lies closer to the points reflecting the process realized using fresh carbons with the greatest oxidizer dose.

This is in line with the results of the conducted analytic studies [36], which showed that-similarly as it the presence of fresh activated carbons-following the introduction of the activated carbon F-200S (with the lowest sorption capacity) to the system comprising dye-hydrogen peroxide, the solution decolorization is accelerated, compared to the results obtained through sorption. However, these changes are not as significant as those obtained in the presence of regenerated activated carbon F-200R, where complete solution decolorization was achieved already after 120 min, at the hydrogen peroxide concentration of 3740 mgH_2_O_2_/L, similarly as in the case of WDex.

## 4. Conclusions

The obtained relations between the parameters in the Freudlich and Langmuir models confirm in most cases that it is possible to develop universal models in which the kinetics of the sorption process is estimated based on the characteristics of the carbons. The conducted analyses show that there is a strong correlation between the pseudo-first and second-order kinetic parameters, and the dose of the oxidizer (H_2_O_2_) as well as the characteristics of the carbons: specific surface area (S), methylene number (MBN), dechlorination half-length (De_hl_). The conducted laboratory studies showed the key influence of the surface area (S) of active carbon and the dose of oxidizer (H_2_O_2_) on the course of the pollutant removal process in the aqueous environment. For example, an increase in the S value from 750 m^2^ to 850 m^2^ (phenol red) assuming a dose of H_2_O_2_ = 800 mg leads to a decrease in the removal time (required removal ratio of 90%) by 29.10%. Increasing the oxidizer dose to the value of H_2_O_2_ = 3200 mg indicates that an increase in S in the range of 750–800 m^2^ leads to a decrease of the minimum time required to removal the dye by 24.01% (removal ratio 90%). The addition of an oxidizer into the system leads to an increase in the value of the constants rate. For De_hl_ = 4.5 and S = 850 m^2^, the constant rate is k_2_ = 0.0105 (phenol red) k_2_ = 0.011 (crystal violet). The addition of the oxidizer in the amount of H_2_O_2_ = 1600 mg leads to an increase in their values to k_2_ = 0.012 and k_2_ = 0.023. This leads to a required degree of removal of pollutants within a shortened time. It was also found that the smaller the surface of the carbon (S), the greater the influence of the oxidizer on the dynamics of the process of dye removal from the aqueous solution. For example, in the case of S = 800 m^2^ and H_2_O_2_ = 1600 mg, 90% dye removal (crystal violet) can be achieved with time shortened by 57% (compared to the system without oxidizer). In turn, for S = 900 m^2^, the same effect of dye removal can be obtained in time shortened by 44.53%. Dechlorination half-length (De_hl_) also has a significant impact on the dynamics of the process. The increase in the De_hl_ value leads to a decrease in the rate of pollution removal. For example, for S = 850 m^2^, the increase in De_hl_ from 4.5 to 4.7 leads to an extension of the removal time of crystal violet from aqueous solution by 17.58% (for removal ratio 90%). For S = 850 m^2^, the increase in De_hl_ from 4.5 to 4.7 results in an extension of the removal time of phenol red from solution by 7.96% (removal ratio 90%). The aforementioned correlations give the assumption for further modelling and process optimization.

The visualization of the similarity levels for the matrix containing the data related to the properties of the applied activated carbons together with oxidizer as well as the kinetic parameters of dye removal based on the multidimensional scaling method (MDS), indicates that the similarity of the spend carbons to the general characteristics describing fresh carbons increases with the oxidizer dose. The highest dose of the oxidizer used (7500 mg H_2_O_2_/L cause that the similarities between the analyzed results of dye removal by all the carbons used (WDex, WG12, F-200S, F-200R) are much greater than in the case of the similarity between the same applied carbons with a high dose oxidizer and no oxidizer.

The developed models allow for analyzing the influence of the specific surface of activated carbon and applied different doses of the oxidizer on the change of the time of pollutants removal from aqueous solution at the required degree. The above-mentioned models also allow for analyzing the influence of the oxidizer dose increase for given surfaces of activated carbons on the decrease of the time of pollutants removal from the aqueous solution until the assumed degree achieved. The models can be also applied for determination of the impact of the change in the De_hl_ value on the time of pollutants removal for the assumed S values and the oxidizer dose.

The models developed on the basis of the analysis of the correlation between the kinetic parameters of the pollutant removal from the aqueous solution and the characteristics of the carbons together with the doses of the oxidizer used, are universal in nature. They allow for the prediction of simultaneous sorption and oxidation processes, taking into account the diversity of physicochemical properties of the sorbent, various adsorbates as well as the type and dose of the oxidizer. Thus, it will enable to optimize the selection of a sorbent and oxidizer used simultaneously for a given group of pollutants which allows to avoid the costly and time-consuming laboratory tests in order to adapt the process to the changing technological parameters.

## Figures and Tables

**Table 1:** Coefficients of the Freundlich and Langmuir isotherms in relations to the adsorption of phenol red and crystal violet onto the investigated activated carbons.

Activated Carbon	Crystal Violet						Phenol Red					
	Freundlich Isotherm			Langmuir Isotherm			Freundlich Isotherm			Langmuir Isotherm		
	K_F_	n	R^2^	a_m_	K_L_	R^2^	K_F_	n	R^2^	a_m_	K_L_	R^2^
**WDex**	9.73	1.92	0.956	50.00	0.33	0.997	6.21	1.54	0.934	83.33	0.67	0.968
**WG-12**	6.30	3.70	0.933	14.29	1.00	0.996	3.15	1.54	0.996	58.82	0.04	0.992
**F-200R**	4.79	3.23	0.996	12.99	0.45	0.994	0.18	0.98	0.706	9.52	0.09	0.894
**F-200S**	1.00	1.18	0.989	58.82	0.01	0.604	0.15	1.15	0.777	43.48	0.002	0.029

**Table 2:** Kinetic parameters of crystal violet and phenol red removal in the presence of hydrogen peroxide and activated carbon.

No.	Procedure	Crystal Violet				Phenol Red			
		First-Order Reaction		Second-Order Reaction		First-Order Reaction		Second-Order Reaction	
		k_1_	R^2^	k_2_	R^2^	k_1_	R^2^	k_2_	R^2^
1	WDex	9.29 × 10^−3^	0.973	1.50 × 10^−2^	0.986	1.03 × 10^−2^	0.965	1.73 × 10^−2^	0.994
2	WDex + 1500 mgH_2_O_2_/L	2.95 × 10^−2^	0.998	6.74 × 10^−2^	0.951	1.03 × 10^−2^	0.965	3.46 × 10^−2^	0.991
3	WDex + 3750 mg H_2_O_2_/L	4.42 × 10^−2^	0.995	12.52 × 10^−2^	0.961	2.23 × 10^−2^	0.968	4.84 × 10^−2^	0.998
4	WDex + 7500 mg H_2_O_2_/L	6.00 × 10^−2^	0.996	18.03 × 10^−2^	0.975	2.26 × 10^−2^	0.966	4.23 × 10^−2^	0.973
5	WG-12	5.47 × 10^−3^	0.852	7.98 × 10^−3^	0.941	8.22 × 10^−3^	0.933	1.29 × 10^−2^	0.994
6	WG-12 + 1500 mg H_2_O_2_/L	8.92 × 10^−3^	0.952	1.44 × 10^−3^	0.991	9.98 × 10^−3^	0.859	1.71 × 10^−2^	0.977
7	WG-12 + 3750 mg H_2_O_2_/L	1.30 × 10^−2^	0.951	2.40 × 10^−2^	0.99	1.09 × 10^−2^	0.880	1.92 × 10^−2^	0.988
8	WG-12 + 7500 mg H_2_O_2_/L	1.52 × 10^−2^	0.974	2.93 × 10^−2^	0.983	1.16 × 10^−2^	0.875	2.08 × 10^−2^	0.988
9	F-200R	6.88 × 10^−3^	0.760	1.05 × 10^−2^	0.900	3.62 × 10^−3^	0.720	4.61 × 10^−3^	0.828
10	F-200R + 1500 mg H_2_O_2_/L	1.25 × 10^−2^	0.960	2.26 × 10^−2^	0.988	4.31 × 10^−3^	0.707	5.73 × 10^−3^	0.835
11	F-200R + 3750 mg H_2_O_2_/L	1.55 × 10^−2^	0.989	2.97 × 10^−2^	0.966	4.66 × 10^−3^	0.693	6.33 × 10^−3^	0.835
12	F-200R + 7500 mg H_2_O_2_/L	2.09 × 10^−2^	0.995	4.38 × 10^−2^	0.955	4.01 × 10^−3^	0.821	5.20 × 10^−3^	0.901
13	F-200S	4.70 × 10^−3^	0.611	6.41 × 10^−3^	0.759	1.60 × 10^−3^	0.880	1.78 × 10^−3^	0.894
14	F-200S + 1500 mg H_2_O_2_/L	5.76 × 10^−3^	0.810	8.20 × 10^−3^	0.891	1.77 × 10^−3^	0.715	2.01 × 10^−3^	0.774
15	F-200S + 3750 mg H_2_O_2_/L	6.92 × 10^−3^	0.907	1.03 × 10^−2^	0.944	1.45 × 10^−3^	0.524	1.61 × 10^−3^	0.584
15	F-200S + 7500 mg H_2_O_2_/L	7.81 × 10^−2^	0.939	1.20 × 10^−2^	0.952	1.83 × 10^−3^	0.592	2.08 × 10^−3^	0.670

**Table 3:** Spearman’s rank correlation coefficient between particular characteristics of the analyzed activated carbons.

Variables	De_hl_	MBN	IN	DetN	Cont. ash	Cont. s. sol	S	Vp	pH
De_hl_	**1.00**	**−0.54**	**−0.89**	**−0.41**	**−0.59**	−0.15	**−0.66**	**−0.58**	**−0.88**
MBN	**−0.54**	**1.00**	**0.71**	**0.67**	**0.64**	−0.11	**0.66**	**0.65**	**0.63**
IN	**−0.89**	**0.71**	**1.00**	**0.58**	**0.69**	0.20	**0.82**	**0.74**	**0.92**
DetN	**−0.41**	**0.67**	**0.58**	**1.00**	0.09	**0.59**	**0.55**	**0.38**	**0.62**
Cont. ash	**−0.59**	**0.64**	**0.69**	0.09	**1.00**	**−0.49**	**0.79**	**0.86**	**0.56**
Cont.s.sol	−0.15	−0.11	0.20	**0.59**	**−0.49**	**1.00**	0.06	−0.15	0.35
S	**−0.66**	**0.66**	**0.82**	**0.55**	**0.79**	0.06	**1.00**	**0.90**	**0.77**
Vp	**−0.58**	**0.65**	**0.74**	**0.38**	**0.86**	−0.15	**0.90**	**1.00**	**0.70**
pH	**−0.88**	**0.63**	**0.92**	**0.62**	**0.56**	0.35	**0.77**	0.70	**1.00**

S—specific surface area (m^2^/g), V_p_—pore volume (cm^3^/g), D_ehl_—dechlorination half-length (cm), IN—iodine number (mg/g), MBN—methylene number (cm^3^), Cont.s.sol.—soluble substance content (%), DetN-detergent number, Cont. ash—ash content (%), pH—pH of the aqueous extract, bolded text-statistically significant correlations.

**Table 4:** Dependences between selected activated carbon parameters and the values of coefficients in the Freundlich and Langmuir isotherm models.

Correlated Parameters	Mathematical Notation of the Equation	Statistical Measures		
	y	r	p	R^2^
	Freundlich Isotherm			
K_F_ (S) (crystal violet)	−14.3513 + 0.0223x	0.9466	0.0534	0.8961
k_F_ (S) (phenol red)	−12.3952 + 0.0178x	0.9464	0.0536	0.8956
n (Cont.s.sol) (crystal violet)	−0.0947 + 1.137x	0.9027	0.0973	0.8150
n (S) (phenol red)	−0.1331 + 0.001x	0.8777	0.1223	0.7703
-	**Langmuir Isotherm**			
K_L_ (Cont.ash) (phenol red)	−0.348 + 0.0493x	0.9915	0.0085	0.9830
a_m_ (MBN) (phenol red)	−86.1458 + 4.574x	0.8153	0.1847	0.6648

**Table 5:** Spearman’s rank correlation between the mean concentration of crystal violet as well as the characteristics of activated carbon and the dose of employed oxidizer.

Variables	H_2_O_2_	De_hl_	MBN	IN	S	c_0_
H_2_O_2_	1.00	−0.15	0.12	0.15	0.08	**−0.55**
De_hl_	−0.15	1.00	−0.54	−0.89	−0.66	**0.84**
MBN	0.12	−0.54	1.00	0.71	0.66	**−0.47**
IN	0.15	−0.89	0.71	1.00	0.82	**−0.84**
S	0.08	−0.66	0.66	0.82	1.00	**−0.80**
c_av_	−0.55	0.84	−0.47	−0.84	−0.80	**1.00**

Bolded text—statistically significant correlations; c_av_—average dye concentration.

**Table 6:** Spearman’s rank correlation between the mean concentration of phenol red as well as the characteristics of activated carbon and the dose of employed oxidizer.

Variables	H_2_O_2_	De_hl_	MBN	IN	S	c_av_
H_2_O_2_	1.00	−0.15	0.12	0.15	0.08	**−0.6**
De_hl_	−0.15	1.00	−0.54	−0.89	−0.66	**0.87**
MBN	0.12	−0.54	1.00	0.71	0.66	−0.39
IN	0.15	−0.89	0.71	1.00	0.82	**−0.75**
S	0.08	−0.66	0.66	0.82	1.00	**−0.61**
c_av_	−0.6	0.87	−0.39	−0.75	−0.61	**1.00**

Bolded text—statistically significant correlations; c_av_—average dye concentration.

**Table 7:** Values of correlation coefficients between the characteristics of activated carbons and the kinetic parameters (pseudo-first/second order) for crystal violet.

Variables	k_1_	k_2_
S	**0.645**	**0.546**
Cont.s.sol	−0.279	−0.376
MBN	**0.673**	**0.546**
De_hl_	**−0.621**	**−0.546**
H_2_O_2_	**0.668**	**0.594**

Bolded text—statistically significant correlations.

**Table 8:** Values of correlation coefficients between the characteristics of activated carbons and the kinetic parameters (pseudo-first/second order) for phenol red.

Variables	k_1_	k_2_
S	**0.922**	**0.946**
Cont.s.sol	−0.049	−0.121
MBN	**0.922**	**0.946**
De_hl_	**−0.922**	**−0.946**
H_2_O_2_	**0.461**	**0.398**

Bolded text—statistically significant correlations.

## Supplementary Materials

The following are available online at https://www.mdpi.com/1996-1944/13/19/4220/s1, Table S1: Physicochemical characteristics of activated carbons selected for investigations, Table S2: Probability value (characteristics of activated carbons), Table S3: Probability value (average dye concentration), Table S4: Probability value (reaction rate constants in the pseudo-first and pseudo-second order equations.
