# Supplementary material for: Modeling and Optimization of Pollutants Removal during Simultaneous Adsorption onto Activated Carbon with Advanced Oxidation in Aqueous Environment
Source: Materials (Basel). 2020 Sep 23;13(19):4220. doi: 10.3390/ma13194220 (PMC7579614; doi:10.3390/ma13194220)
Supplement: Supplementary file 1 [file materials-13-04220-s001.pdf]

materials

MDPI

Supplementary Materials

Modeling and Optimization of Pollutants Removal during Simultaneous Adsorption onto Activated Carbon with Advanced Oxidation in Aqueous Environment

Lidia Dąbek [1], Anna Picheta-Oleś [2], Bartosz Szeląg [1,*], Joanna Szulżyk-Cieplak [3] and Grzegorz Łagód [4,*]

[1] Faculty of Environmental, Geomatic and Energy Engineering, Kielce University of Technology, Tysiąclecia Państwa Polskiego 7, 25-314 Kielce, Poland; ldabek@tu.kielce.pl

[2] Department of Environment and Waste Management, Marshal's Office of the Świętokrzyskie Voivodeship, IX Wieków Kielc 3, 25-516 Kielce, Poland; anna.picheta-oles@sejmik.kielce.pl

[3] Faculty of Fundamentals of Technology, Lublin University of Technology, Nadbystrzycka 38, 20-618 Lublin, Poland; j.szulzyk-cieplak@pollub.pl

[4] Environmental Engineering Faculty, Lublin University of Technology, Nadbystrzycka 40B, 20-618 Lublin, Poland

* Correspondence: bszelag@tu.kielce.pl (B.S.); g.lagod@pollub.pl (G.Ł.); Tel.: +48-4134-3473 (B.S.); +48-81-538-4322 (G.Ł.)

Received: 29 August 2020; Accepted: 21 September 2020; Published: date

Table S1. Physicochemical characteristics of activated carbons selected for investigations.

Carbon	S	V_p	D_{ehl}	IN	MBN	DetN	Cont.s.sol.	Cont. ash	pH
WDex	1050	0.95	5	990	36	20.6	1.07	20.64	8.6
WG-12	980	0.89	3	1230	32	30.2	2.48	8.85	9.6
F-200S	720	0.72	7	710	24	12.7	1.16	7.28	4.5
F-200R	800	0.86	6	770	26	15.7	1.74	7.84	7.2

S—specific surface area (m^2/g), V_p—pore volume (cm^3/g), D_{ehl}—dechlorination half-length (cm), IN—iodine number (mg/g), MBN—methylene number (cm^3), Cont.s.sol.—soluble substance content (%), DetN.—detergent number, Cont. ash—ash content (%), pH—pH of the aqueous extract.

In Table S2, Table S3 and Table S4, respectively, determined values of the test probability are given for the correlation between the selected characteristics of the activated carbons, average dye concentration (c_{av}) and the reaction rate constants in the pseudo-first and pseudo-second order equations.

Table S2. Probability (p) value (characteristics of activated carbons).

Variables	D_{ehl}	MBN	IN	DetN	Cont. ash	Cont.s.sol	S	V_p	pH
D_{ehl}	**0.00000**	**0.03124**	**0.00433**	**0.04436**	**0.02665**	0.07567	**0.02064**	**0.02755**	**0.00493**
MBN	-	**0.00000**	**0.01664**	**0.01982**	**0.02230**	0.08108	**0.02064**	**0.02147**	**0.02315**
IN	-	-	**0.00000**	**0.02755**	**0.01821**	0.06912	**0.00873**	**0.01436**	**0.00259**
DetN	-	-	-	**0.00000**	0.08385	**0.02665**	**0.03031**	**0.04763**	**0.02401**
Cont. ash	-	-	-	-	**0.00000**	**0.03609**	**0.01077**	**0.00616**	**0.02938**
Cont.s.sol	-	-	-	-	-	**0.00000**	0.08808	0.07567	0.05099
S	-	-	-	-	-	-	**0.00000**	**0.00374**	**0.01218**
V_p	-	-	-	-	-	-	-	**0.00000**	**0.01742**
pH	-	-	-	-	-	-	-	-	**0.00000**

where $p \leq 0.05$-statistically significant correlations (bold in table).

Table S3. Probability value (average dye concentration).

Variables	c_{av} (Crystal Violet)	c_{av} (Phenol Red)
H_2O_2	**0.026**	**0.018**
De_{hl}	**0.006**	**0.008**
MBN	**0.047**	0.051
IN	**0.006**	**0.013**
S	**0.008**	**0.032**

c_{av}—average dye concentration (mg/L). where: $p \leq 0.05$-statistically significant correlations (bold in table).

Table S4. Probability value (reaction rate constants in the pseudo-first and pseudo-second order equation

Variables	Crystal Violet		Phenol Red	
	k_1	k_2	k_1	k_2
S	**0.02**	**0.031**	**0.004**	**0.003**
Con.s.sol.	0.067	0.051	0.074	0.064
MBN	**0.023**	**0.031**	**0.004**	**0.003**
De_{hl}	**0.022**	**0.031**	**0.004**	**0.003**
H_2O_2	**0.017**	**0.029**	**0.038**	0.051

where: $p \leq 0.05$—statistically significant correlations (bold in table).

© 2020 by the authors. Submitted for possible open access publication under the terms and conditions of the Creative Commons Attribution (CC BY) license (http://creativecommons.org/licenses/by/4.0/).
